# Unilateral acute cataract after facial aesthetic procedure: a case report

**DOI:** 10.5935/0004-2749.2023-0248

**Published:** 2024-09-16

**Authors:** Rafael Norio Makibara, Leonardo Pimenta de Oliveira, Marcela Drummond de Andrade Rocha

**Affiliations:** 1 Departamento de Oftalmologia, Hospital Santa Luzia, Fundação Colombo Spínola, Salvador, BA, Brazil

**Keywords:** Cosmetic techniques, Skin aging, Rejuvenation, Ultrasonic therapy/adverse effects, High-intensity focused ultrasound ablation/methods, Cataract/etiology, Phacoemulsification, Lens implantation, intraocular, Visual acuity, Humans, Middle age, Female, Case reports

## Abstract

Aging and face sagging have many causes, and various techniques are used for treatment, including noninvasive procedures, such as focused ultrasound, which uses the principle of collagen regeneration by coagulative necrosis of the dermis layers using radiofrequency, but this procedure has complications. We reported a case of a 54-year-old female patient who complained of poor visual acuity in her right eye three days after a focused ultrasound facial aesthetic procedure, with the best visual acuity of 20/60. Biomicroscopy of the right eye revealed an acute cataract with three points of fibrosis extending from the posterior to the anterior capsule. The patient underwent phacoemulsification surgery with visual rehabilitation and improved vision of 20/20. We hypothesized that the occurrence of acute cataract was related to the inappropriate use of focused ultrasound.

## INTRODUCTION

Aging and face sagging have various causes, and several treatment options are currently available, including minimally invasive procedures (without skin incision) that are preferable to surgical procedures, mainly because of the more natural results and faster recovery^(1)^. One example is the application of radiofrequency-focused ultrasound, which uses the principle of thermal heating (approximately 65°C) controlled by energy (between 0.4 and 1.2 J/mm²) and frequency (between 4 and 10 MHz), which can induce small thermal coagulation points at a depth of up to 5 mm within the middle and deep reticular layers of the epidermis and dermis. This mechanism not only leads to local coagulation but also causes the collagen chains to contract into more stable forms.

Two ultrasound types are used: microfocused ultrasound (MFU), which is the most suitable for facial areas with mild to moderate sagging, and high-intensity focused ultrasound (HIFU), which is suitable for tumor and adipose tissue ablation and body contouring^(2)^. In the periorbital area, these devices improve the appearance of the eyelid region. To avoid intraocular lesions, the site should be limited to the orbital portion of the orbicularis muscle and bone^(3)^. It is minimally invasive and relatively safe. However, some devices do not provide real-time images of the application site, making visualization of the anatomical structures difficult, which could to complications, mainly due to thermal (e.g., burns, peeling, and hypersensitivity), vascular (hematoma and ecchymosis), and nerve damage (nerve paralysis and paresis and its affected innervation)^(4)^. MFU is most commonly used to lift the eyebrows and should be applied to the lateral part of the forehead, including the two lateral thirds, with a depth not exceeding 3 mm^(5)^. We report a case of unilateral acute cataract that we hypothesized to be due to the incorrect or inadvertent use of an aesthetic procedure with ultrasound on drooping eyelids.

## CASE REPORT

**Figure 1:**
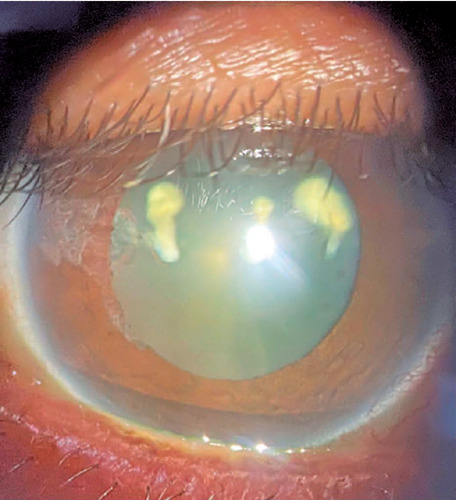
Biomicroscopy of the right eye (OD) with temporal iris atrophy and cataract with three points of fibrosis from the anterior to the posterior lens capsule.

**Figure 2:**
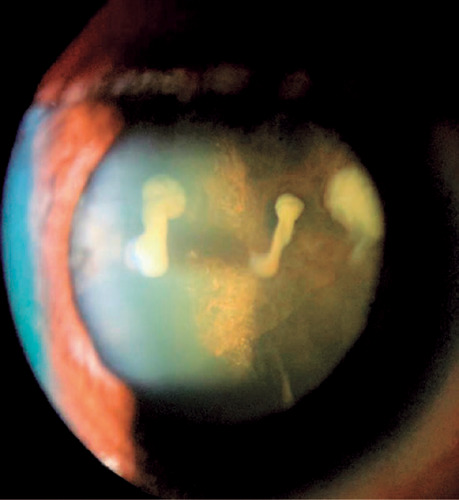
Biomicroscopy of the right eye (OD) with cataract and three points of fibrosis from the anterior to the posterior lens capsule.

A 54-year-old female patient consulted an ophthalmologist complaining of blurred vision and low visual acuity (VA) in her right eye (OD) for 3 days. She reported that the symptoms had started after a right periorbital-focused ultrasound cosmetic procedure performed for droopy eyelids secondary to her peripheral facial palsy in 2009. She gave no further details about the device type or parameters used, but she was not instructed to wear eye protection. The patient had an unremarkable medical or ophthalmologic history and reported good vision in both eyes (BE) before the procedure. On ophthalmologic examination, the patient had better Snellen-corrected VA in the right eye (OD) at 20/60 and left eye (OS) at 20/20. In RE, biomicroscopy showed corneal edema, temporal iris atrophy, and lens opacification with three points of fibrosis from the posterior to the anterior capsule (Figures 1 and 2), and fundoscopy showed cloudy media due to the cataract, making assessment challenging. The intraocular pressure was 10 mmHg at BE, and an ophthalmologic examination of the OS showed no changes.

**Figure 3:**
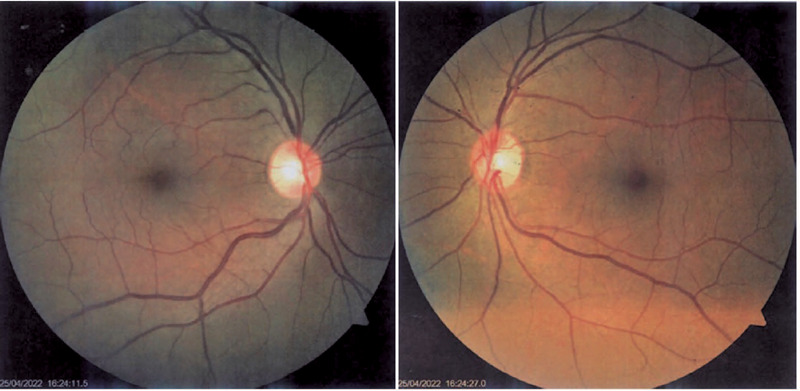
Fundus photograph of both eyes (BE) with normal findings.

**Figure 4:**
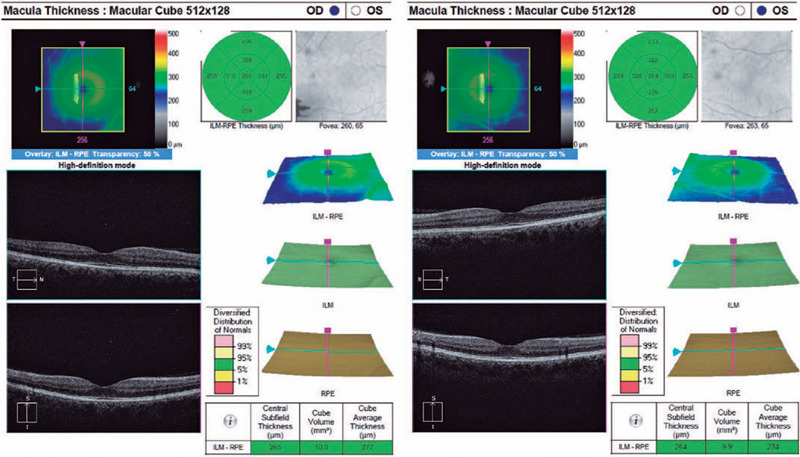
Optical coherence tomography (OCT) of both eyes (BE) shows normal examination with preserved retinal layers.

We performed implantation of an intraocular lens based on our diagnostic hypothesis of a traumatic cataract. On the first postoperative day (POD), the patient’s OD biomicroscopy revealed a nonhyperemic eye, persistent corneal edema, temporal iris atrophy, a wide and shaped anterior chamber, and a topical intraocular lens. On POD 14, the Snellen-corrected VA was 20/20 (-0.75 to -0.75 150°) in OD. To confirm that the patient had no ocular pathology, we performed retinography (Figure 3) and optical coherence tomography (OCT) of the macula BE (Figure 4).

## DISCUSSION

Focused ultrasound is used for aesthetic purposes because of its noninvasiveness. They are based on the principle of using energy (radiofrequency) to cause thermal coagulation of the tissue, transforming collagen fibers in the subcutaneous layer into more stable forms^(6)^. Due to the occurrence of potential complications, a qualified professional familiar with the anatomy who can understand ultrasound images should perform the procedure because the safety and effectiveness depend on the correct application and depth of the region to be treated^(7)^. Lizzi et al. demonstrated in vivo that high-frequency ultrasound can produce cataracts, proportional to the amount of energy and time used^(8)^. Some reports showed that HIFU damages the cornea (stromal opacification), iris (accommodative spasm), and lens (cataract) due to the duration of application and choice of probe, as well as failure to wear eye protection^(5^,^9)^. Ikoma et al. reported a similar case of acute drop cataract in the LE that developed 7 days after a patient underwent an ultrasound aesthetic eyelid treatment, but with unknown energy and without the use of a protective device^(10)^.

The patient’s case showed a cataract that probably developed because of the improper use of eyelid ultrasound, which is typical of heat-induced eye trauma. Most cataracts develop slowly and painlessly. Traumatic cataracts (blunt or penetrating), whether from electric shock, chemical burns, or radiation, acutely lead to crystalline opacification within days or weeks^(9)^. The clinical history, eye examination findings, and the procedure performed confirmed that the changes may have been caused by inadequate performance of the procedure, which must be customized for each patient based on the indication, i.e. the ultrasound type, energy, frequency, transducer type, region treated, and eye protection device use^(10)^.

The patient underwent phacoemulsification surgery to improve her vision. Fortunately, she achieved full vision and quality of life after the procedure. This report shows that aesthetic eyelid procedure has complications and that caution is required to achieve the desired goal. The indication, ultrasound type, parameters, and eye protection during the procedure are fundamental to minimize this type of risk.
